# Risk factor analysis for above-knee amputation in patients with periprosthetic joint infection of the knee: a case-control study

**DOI:** 10.1186/s12891-021-04757-w

**Published:** 2021-10-18

**Authors:** Franziska Eckers, Christoph J. Laux, Sebastian Schaller, Martin Berli, Yvonne Achermann, Sandro F. Fucentese

**Affiliations:** 1 Department of Orthopaedics, Balgrist University Hospital, University of Zurich, Forchstrasse 340, 8008 Zurich, Switzerland; 2 Department of Infectious Diseases and Hospital Epidemiologie, University Hospital Zurich, University of Zurich, Rämistrasse 100, 8091 Zurich, Switzerland

**Keywords:** Periprosthetic joint infection, PJI, Arthroplasty, Total knee arthroplasty, TKA, Above-knee amputation, Risk factors

## Abstract

**Background:**

Periprosthetic joint infection (PJI) is a severe complication following knee arthroplasty**.** Therapeutic strategies comprise a combination of surgical and antibiotic treatment modalities and aim to eradicate the infection. Sometimes control of the disease can only be attained by above-knee amputation (AKA). While a vast amount of literature exists illuminating predisposing factors for PJI, risk factors favoring the endpoint AKA in this context are sparsely known.

**Methods:**

The purpose of this investigation was to delineate whether patients with PJI of the knee present specific risk factors for AKA. In a retrospective case-control study 11 cases of PJI treated with AKA were compared to 57 cases treated with limb salvage (LS). The minimum follow-up was 2 years. Comorbidities, signs and symptoms of the current infection, factors related to previous surgeries and the implant, microbiology, as well as therapy related factors were recorded. Comparative analysis was performed using student’s t-test, chi-square test or Fisher’s exact test. Binary differences were calculated using odds ratio (OR). Reoperation frequency was compared using Mann-Whitney U test. In-depth descriptive analysis of 11 amputees was carried out.

**Results:**

A total of 68 cases aged 71 ± 11.2 years were examined, 11 of which underwent AKA and 57 had LS. Severe comorbidities (*p* = 0.009), alcohol abuse (*p* = 0.015), and preoperative anemia (*p* = 0.022) were more frequently associated with AKA. Preoperative anemia was found in all 11 amputees (100%) and in 33 of 57 LS patients (58%) with an average preoperative hemoglobin of 99.9 ± 15.1 g/dl compared to 118.2 ± 19.9 g/dl (*p* = 0.011). No other parameters differed significantly. AKA patients underwent a median of eight (range 2–24) reoperations, LS patients a median of five (range 2–15).

**Conclusion:**

Factors potentially influencing the outcome of knee PJI are diverse. The indication of AKA in this context remains a rarity and a case-by-case decision. Patient-intrinsic systemic factors such as alcohol abuse, severe comorbidities and preoperative anemia may elevate the individual risk for AKA in the setting of PJI. We recommend that anemia, being a condition well amenable to therapeutic measures, should be given special consideration in management of PJI patients.

**Trial registration:**

This study was registered with Kantonale Ethikkommission Zürich, (BASEC-No. 2016–01048).

## Introduction

Periprosthetic joint infection (PJI) is a rare but severe complication following knee arthroplasty with an overall incidence of approximately 1–2% [1–3]. It is one of the most common indications for revision surgery [4, 5]. Therapeutic strategies comprise a combination of surgical and antibiotic treatment modalities and aim to eradicate the infection while salvaging life and limb. Surgically, there are essentially two different methods to employ: (1.) irrigation, debridement and implant retention with exchange of the polyethylene liner (often referred to as DAIR (debridement, antibiotics and implant retention)), (2.) (staged) exchange arthroplasty, each complemented by targeted antibiotic therapy. The choice of strategy is subject to several decisive factors including timing, host profile, and causative microorganism. At our institution, the first option is employed as standard of care in cases of early postoperative or acute hematogenous infection without signs of implant loosening (within 30 days of prosthesis implantation or duration of symptoms < 3 weeks) [6–8]. However, failure rates of treatment with implant retention are reported to be high. Careful patient selection should be emphasized [9, 10]. As to exchange arthroplasty for treatment of delayed or chronic PJI (beyond 30 days of prosthesis implantation or duration of symptoms > 3 weeks), the choice between single-stage and two-stage procedures is currently controversially discussed [11]. While the former is now viewed as a “reasonable option for the treatment of PJI in circumstances where effective antibiotics are available” [8], we have up to this point been favoring the latter as the standard treatment in this context with a reported success rate of 92.5% according to a 2019 by Pangaud et al. [12].

In certain patients, however, the above-mentioned treatment strategies fail. Some of these patients qualify for knee arthrodesis, while in others, control of the disease can only be attained by above-knee amputation (AKA). At our institution we recently observed an increased rate of AKA for treatment of knee PJI, drawing focus to the subject. On inspection we found that 11 out of 129 cases underwent AKA – thus 8.5% - during an observation period of 11 years. For comparison, based on data retrieved from the U.S. Medicare Inpatient Claims Database, a 2017 survey by Son et al. that included 44,466 cases of PJI of the knee documented between 2004 and 2015, reported as many as 4% of patients having to undergo AKA in this context, and even a 7% risk for AKA at 10 years from diagnosis of PJI [13]. The implications of the procedure are grave. Functional outcome is generally poor [14, 15], while postoperative mortality is reported to be high, ranging from 50 to 60% at 1 year postoperatively [16, 17]. Those who survive often forfeit their independence and are henceforth reliant on assistance [14, 18].

There is a vast amount of literature regarding the subject of PJI, specifically as to predisposing factors that may increase a patient’s risk for developing this devastating complication [3, 19]. As such, a series of comorbid conditions as well as some therapy related factors are known. In which of the affected patients, however, do we fail to preserve the limb while treating the PJI? Previously conducted studies looking at PJI treatment failure were able to identify the presence of certain causative bacteria such as S. aureus [20] and Enterococci [21] to be associated with poor outcomes. Obesity, liver cirrhosis, gram-negative organism, and the presence of sinus tract have been found to be risk factors for failure after hip reimplantation arthroplasty [22] .

Failure of treatment of PJI of the knee may ultimately end up in AKA. Within this investigation we aimed to delineate whether or not patients who undergo AKA for treatment of PJI of the knee present specific risk factors, either patient related or associated to our treatment. Are there factors that – once identified – would be either amenable to treatment or that would otherwise influence medical decision-making and action? In addition to answering these study questions, we carried out an in-depth descriptive evaluation of the 11 amputees.

## Methods

### Patient selection

This study was carried out in a university hospital providing subspecialized orthopedic tertiary care. A search inquiry of the institutional database using the appropriate search terms (knee AND septic arthritis/prosthetic joint infection) identified a total of 129 cases who had undergone surgical treatment of knee PJI between 2005 and 2015, thereof 11 with AKA and 118 with limb salvage (LS). As seamless documentation was considered crucial, completeness of patient records was first verified. In this process it was noted that earlier years’ digital patient records were in part incomplete.. While all 11 AKA cases were well documented, the same was not the case for all LS patients. Therefore, in order to form a representative control group, we selected the 66 most recent LS cases, consecutively. Further processing led to the exclusion of nine more cases due to incomplete medical records, ultimately leaving 57 LS fully documented cases for evaluation. Subsequently, by means of a retrospective case-control study, 11 cases of PJI needing AKA were compared to 57 cases of PJI with LS.

### Data acquisition

The minimum follow-up was 2 years, with the first septic revision surgery being defined as point zero. All clinical and laboratory parameters presented were obtained preoperatively to this first surgery, on hospitalization for treatment of PJI, respectively. Patients’ informed consent and the approval of the responsible ethics committee (Kantonale Ethikkommission Zürich, BASEC-No. 2016–01048) were obtained.

The presence of PJI was established by applying the definition of the *International Consensus Group* from 2013, thus by confirming the presence of one of two major criteria (two positive periprosthetic cultures with phenotypically identical organisms or a sinus tract communicating with the joint) or by presence of four minor criteria ((1.) elevated serum C-reactive protein (CRP) and erythrocyte sedimentation rate (ESR), (2.) elevated synovial fluid white blood cell (WBC) count, (3.) elevated synovial fluid polymorphonuclear neutrophil percentage (PMN%), (4.) a single positive culture) [23].

Data acquisition included (1.) demographics and comorbidities represented by the American Society of Anesthesiologists (ASA) classification system [24] and the Charlson comorbidity score [25], (2.) disease related factors including pre- and perioperative clinical and laboratory parameters, the acuity of the symptomatology as well as microbiological aspects, (3.) factors related to previous surgeries and the implant, and, (4.) therapy related factors such as revision strategy, number of surgeries, and details of antibiotic treatment. Surgeries involving the removal, and/or replacement of prosthetic components/cement spacers, as well as temporary or definite arthrodesis were defined as major revisions. All other surgeries, usually washouts addressing only the skin and subcutaneous layer, were defined as minor reoperations.

### Statistical analysis

Subsequently, subgroup analysis was performed. Results of patients with AKA were compared to those of patients with LS. Furthermore, in-depth descriptive analysis of the 11 amputees was carried out.

Descriptive statistics were used to outline patient demographics and characteristics. Continuous variables are presented as mean with standard deviation for normal distribution, and as median with range for non-normal distribution. Normal distribution was tested using Kolmogorow-Smirnov test with Lilliefors adaption. Categorical variables are presented by frequency. Differences between groups were tested with student’s t-test, chi-square test or Fisher’s exact test, as applicable. Binary differences were calculated using odds ratio (OR). Revision and reoperation frequency was compared using Mann-Whitney U test. *P* < 0.05 was considered statistically significant.

## Results

During an 11-year observation period a total of 129 cases of PJI of the knee were surgically treated at our institution, 11 of which received AKA, hence 8.5%. All 11 amputation cases as well as a representative cohort of 57 limb salvage cases were included in this study. Among the latter, seven lower limbs were salvaged by knee arthrodesis, the others by (staged) revision arthroplasty.

Results are presented for the total of examinees with subgroup results added where statistical significance is present. A comprehensive summary of all outcome parameters is illustrated in Table 1*.* Additional comparative subgroup analysis of ASA scores is presented in Table 2. Detailed bacteriological results are displayed in Table 3. Revision and reoperation frequencies are compared in Table 4.

**Table 1:** Comprehensive summery of all outcome parameters with subgroup analysis AKA versus LS

	all patients (***n*** = 68)		AKA (***n*** = 11)		LS (***n*** = 57)		***P***
	n (%)	mean ±	n (%)	mean ±	n (%)	mean ±	
**DEMOGRAPHICS**							
**Gender**							
Female	37 (54)		6 (56)		31 (54)		0.992
Male	31 (46)		5 (45)		26 (46)		
**Patient age at index surgery**		71.0 ± 11.2		67.4 ± 14.3		71.7 ± 10.5	0.249
**BMI (kg/m**^**2**^**)**		30.6 ± 8.2		29.5 ± 11.5		30.8 ± 7.5	0.619
**Smoker**	16 (24)		5 (45)		11 (19)		0.061
**Alcohol abuse**	3 (4)		2 (18)		1 (2)		0.015
**ASA**							
1	0 (0)		0 (0)		0 (0)		0.009
2	27 (40)		1 (9)		26 (46)		
3	36 (53)		10 (91)		26 (46)		
4	5 (7)		0 (0)		5 (9)		
**Charlson Score ≥ 3**	13 (19)		4 (36)		9 (16)		0.112
**DISEASE RELATED FACTORS**							
**Type of infection**							
Acute/early	47 (69)		9 (82)		38 (67)		0.439
Chronic/delayed	18 (26)		2 (18)		16 (28)		
Unknown	3 (4)		0 (0)		3 (5)		
**Joint fistula present**	11 (16)		1 (9)		10 (18)		0.486
**Anemia**^a^	44 (65)		11 (100)		33 (58)		0.022
**Hemoglobin (g/dl)**		115 ± 21		98 ± 15		118 ± 20	0.011
**CRP (mg/l)**		130 ± 113		119 ± 106		132 ± 115	0.736
**WBC (n × 10**^**9**^**/l)**		10 ± 4		9 ± 3		11 ± 5	0.355
**Positive blood cultures**	18 (26)		4 (36)		12 (21)		0.273
**Joint aspirate: leucocyte cell count (n/mm**^**3**^**)**^b^		32,920 ± 33,028		68,030 ± 11,652		21,230 ± 16,329	0.404
**Microbiology:**							
Monomicrobial	41 (60)		7 (64)		34 (60)		0.976
Polymicrobial	19 (28)		4 (36)		15 (26)		0.497
Culture negative	6 (9)		0 (0)		6 (11)		0.260
Incomplete records	2 (3)		0 (0)		2 (4)		
**Resistance pattern**							
Pansensitive	35 (51)		4 (36)		31 (54)		0.148
Resistances present	10 (15)		1 (9)		20 (35)		
Multiresistent	23 (34)		6 (56)		17 (30)		
**IMPLANT/PREVIOUS SURGERY RELATED FACTORS**							
**Knee surgery prior to KA**	17 (25)		3 (27)		14 (25)		0.849
**Patient age at KA implantation**		67.2 ± 11.3		63.6 ± 15.2		67.9 ± 10.5	0.249
**Primary implantation elsewhere**	15 (22)		3 (27)		12 (21)		0.649
**Type of implant**							
Unicompartimental	1 (1)		0 (0)		1 (2)		0.658
PS/CR	53 (78)		7 (64)		46 (81)		0.211
Hinged	11 (16)		3 (27)		8 (14)		0.275
Megaprosthesis	3 (4)		1 (9)		2 (4)		0.409
**Revision prosthesis**	14 (21)		3 (27)		11 (19)		0.549
**TREATMENT RELATED FACTORS**							
**Previous intervention elsewhere**	22 (32)		5 (45)		17 (30)		0.310
**Blood transfusion**	23 (34)		6 (56)		17 (30)		0.122

^a^The world health organization identifies anemia as hemoglobin thresholds of 12.0 g/dl for non-pregnant women and 13.0 g/dl for men

^b^Joint aspiration was carried out in 62 cases

**Table 2:** Analysis of ASA scores

	AKA (***n*** = 11) n (%)	LS (***n*** = 57) n (%)	prevalence of AKA (%)	Odds ratio (OR)	*p* ASA 2 vs. 3 (Fisher’s exact test)
**ASA**					
**2**	1 (9)	26 (46)	3.70	0.12	0.0174
**3**	10 (91)	26 (46)	27.78	11.92	
**4**	0 (0)	5 (9)	0	0

**Table 3:** Bacteriological analysis

	all patients (***n*** = 68)	AKA (***n*** = 11)	LS (***n*** = 57)
	n (%)	n (%)	n (%)
**Monomicrobial**	41 (60)	7 (64)	34 (60)
S. aureus	13 (19)	1 (9)	12 (21)
Coagulase negative staphylococci	16 (24)	2 (18)	14 (25)
Streptococcus species	4 (6)	2 (18)	2 (4)
Enterobacteriaceae			
E. coli	3 (4)	0 (0)	3 (5)
E. cloacae	1 (1)	0 (0)	1 (2)
Anaerobic bacteria			
C. acnes	1 (1)	0 (0)	1 (2)
Corynebacterium striatum	1 (1)	1 (9)	0 (0)
Francisella tularensis	1 (1)	0 (0)	1 (2)
Candida albicans	1 (1)	1 (9)	0 (0)
**Polymicrobial**	19 (28)	4 (36)	15 (26)
**Culture negative**	6 (9)	0 (0)	6 (11)
**Incomplete records**	2 (3)	0 (0)	2 (4)

**Table 4:** Revision and reoperation frequencies, AKA versus LS

	AKA (***n*** = 11)			LS (***n*** = 57)			***P***
	Median	Min	Max	Median	Min	Max	
**Surgeries (total)**	8	2	24	5	2	15	0.194
**Minor reoperation**	4	1	12	2	1	6	0.061
**Major revision**	4	1	13	3	1	9	0.380

### Demographics

Regarding patient demographics the following results were found: Thirty-seven were women and 31 men, with a mean age of 71 ± 11.2 at the time of the index surgery. The mean BMI was 30.6 ± 8.2 kg/m^2^. Sixteen patients (24%) were smokers; smoking tended to be more frequent in the AKA group (5 (45%) vs. 11 (19%); *p* = 0.061). Three patients (4%) suffered from alcohol abuse, in two of which limb salvage failed, associating alcohol abuse with AKA (2 (18%) vs. 1 (2%); *p* = 0.015).

Concerning comorbidities as measured by ASA grading we surgically treated 27 (40%) with ASA 2, 36 (53%) with ASA 3, and 5 (7%) with ASA 4. Comparative subgroup analysis, as presented in Table 2, found one AKA case (9%) and 26 LS cases (46%) to be classed as ASA 2, and ten AKA cases (91%) and 26 LS cases (46%) to be classed as ASA 3. Five LS cases (9%) were classed ASA 4. The OR for ASA 3 patients versus the remainder of patients was 11.92 for AKA versus LS. Fisher’s exact test showed that ASA 3 patients have a significantly higher risk for AKA than ASA 2 patients (*p* = 0.0174). Pearson’s chi-squared test confirmed the severity of comorbidities based on ASA scoring to be higher in the group with AKA (*p* = 0.009). No statistic difference was found when using the Charlson comorbidity score for assessment of severity of comorbidities.

### Disease related factors

Forty-seven cases (69%) suffered of an acute/early, 18 (26%) had a chronic/delayed infection. In three cases (5%) duration or time of appearance of symptoms was not known. No association between timing of disease and choice of index procedure was observed. In 11 cases (16%) a joint fistula was present. Preoperative anemia [26] with hemoglobin levels lower than 120 g/l in women, and lower than 130 g/l in men, respectively, was found in 44 cases (65%) and was significantly more often associated with AKA than with LS (11 (100%) vs. 33 (58%); *p* = 0.022) with an average preoperative hemoglobin level of 99.9 ± 15.1 g/dl in the AKA group, compared to 118.2 ± 19.9 g/dl in the LS group (*p* = 0.011). No other blood parameter differed significantly. Preoperative joint aspiration rendered a mean leucocyte cell count of 32,920 ± 33,028/mm^3^ with granulocytes being the predominant cell type in all aspirates.

Bacteriological testing revealed 41 mono- and 19 polymicrobial infections (60, 28% respectively). Six PJI (9%) were culture negative. In two cases records were incomplete. The most commonly isolated germs were staphylococci (14 *S. aureus*, 17 coagulase-negative Staphylococci (CNS)). Other types of causative bacteria were Streptococci, Enterobacteriaceae such as *E. coli* and *E. cloacae*, and anaerobic bacteria such as *C. acnes* and Corynebacteria. One patient presented with a PJI caused by *Francisella tularensis*, another with a fungal infection caused by *Candida albicans*. A complete list of the identified microorganisms is provided in Table 3. No association between mono- versus polymicrobial infections and type of index procedure was found, neither between antibiotic resistance pattern and index procedure. The small number of pathogens did not allow for statistical analysis of different species.

### Implant / previous surgery related factors

Analysis of factors regarding the type of implant, previous surgeries, and the displayed aspects of treatment did not yield any significant results.

### Revisions and reoperations

As outlined in Table 4, the AKA patients underwent a median of eight (range 2–24) surgeries, involving four (range 1–12) superficial reoperations or washouts and four (range 1–13) major revisions, while the LS patients underwent a median of five (range 2–15) surgeries, involving two (range 1–6) minor reoperations and three (1–9) major revisions. The AKA group tended to have more minor reoperations (*p* = 0.061).

### Eleven cases of PJI treated with AKA

**Fig. 1:**
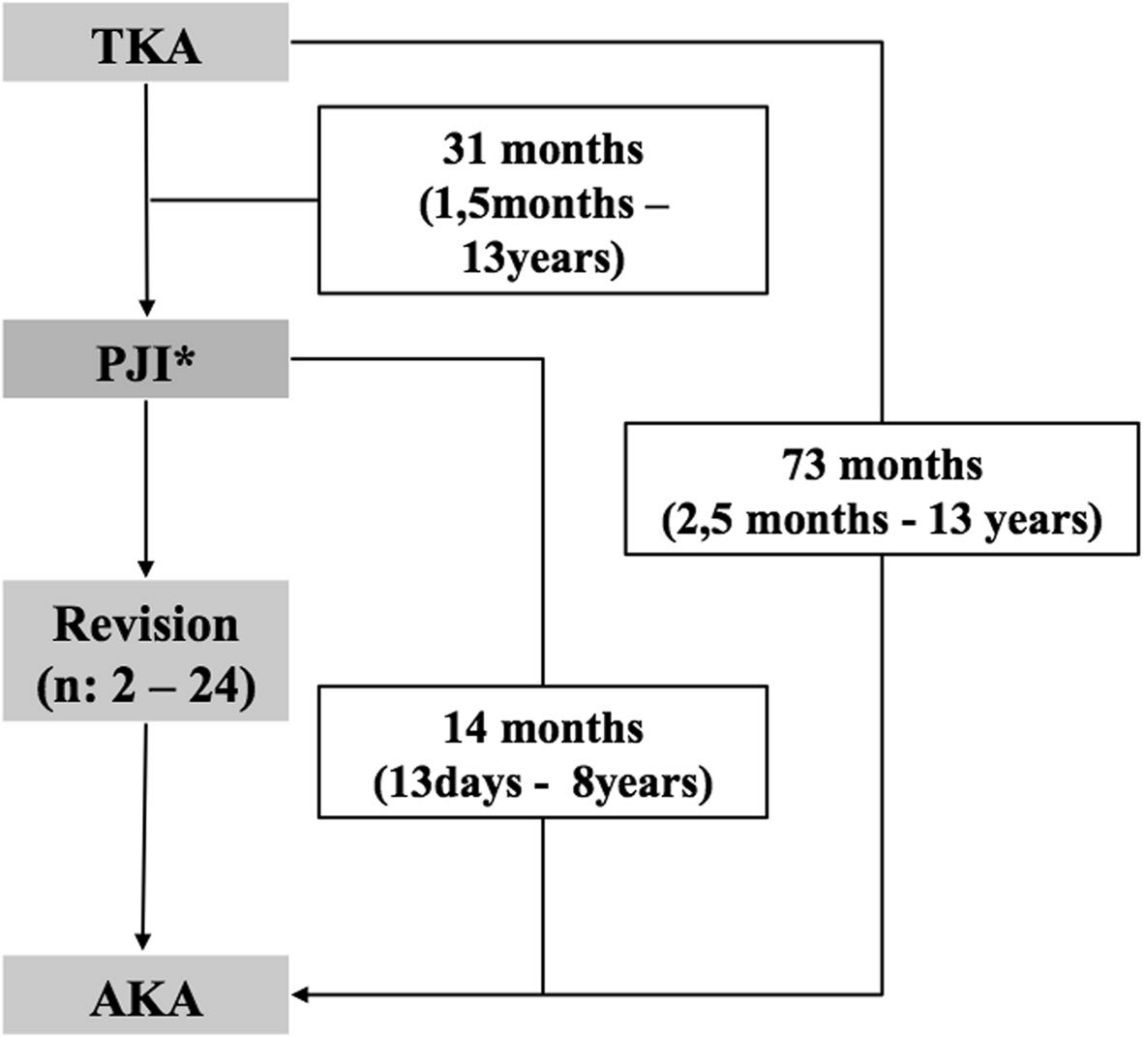
illustrates the chronological sequence of events for the 11 patients having undergone AKA in the context of PJI. * 9 early/acute, 2 chronic/delayed PJI

Figure 1 shows the chronological sequence of events for the 11 examined amputees. A median of 31 months (range 1.5 months – 13 years) passed between total knee arthroplasty (TKA) and diagnosis of PJI. From time of start of treatment at our institution - marked by the first revision surgery - until AKA, time spans of 13 days up to 8 years went by. During this time most patients had to undergo multiple surgeries (median 8, range 2–24). Time between TKA and AKA was a median of 73 months (range 2.5 months – 13 years). The mean age at amputation was 67.4 ± 14.3 years. There were six females and five males. Case eight and eleven are the same person; this patient eventually underwent bilateral AKA in the presence of knee PJI.

As illustrated in Table 5, almost all patients suffered from multiple comorbidities and immunosuppressive conditions such as diabetes, morbid obesity, chronic pulmonary and cardiovascular diseases, and recurrent infections. Three (*8, 10, 11*) suffered from rheumatoid arthritis or chronic arthralgia with long-term treatment with steroids and/or disease-modifying antirheumatic drugs. One patient (*3*) had been diagnosed with myelodysplastic syndrome shortly after implantation of TKA and had therefore been placed under immunosuppressants. One patient (*7*) was a polytoxicomaniac with chronic hepatitis B/C as well as infection with human immune deficiency virus (HIV). Absence of systemic diseases was observed in only one case (*4*); this patient, however, had previously received a megaprosthesis with interpolated gastroc−/soleus-flap for a Gustilo 3C [27] open tibial plateau fracture. He was also one of two patients with documented alcohol abuse. Regarding the type of infection, nine cases were classified as early or acute PJI and two as chronic or delayed PJI. All patients were anemic at time of admission to hospital with a mean hemoglobin level of 97.3 ± 15.1 g/dl. Four PJI were polymicrobial, three were caused by Staphylococci (2 coagulase negative Staphylococci (CNS), 1 *S. aureus*), two by Streptococci, one by anaerobic bacteria and one by *C. albicans*. The latter was found in the aforementioned patient with HIV.

**Table 5:** Overview 11 patients with AKA for PJI

	Age at AKA	Gender	BMI (kg/m^**2**^)	Smoker	Alcohol abuse	ASA	Relevant comorbidities, patient particularities	Type of infection	Initial strategy	Anemia	Hb (g/dl)	Microrga-nism(s)	Surgeries (total)	Indication for AKA
**1**	86	f	44.4	no	no	3	DM, AH, morbid obesity, severe malnutrition, inguinal mykosis, CRI, ncnc anemia, UTI	chronic/ delayed	AKA	yes	109	CNS	3	Chronic TKA-dislocation and PJI. Ongoing deterioration in spite of TKA explantation and antibiotic treatment. Low functional demand.
**2**	77	f	51.9	no	no	3	AH, AF, morbid obesity, severe malnutrition, COPD, s/p ARI, inguinal mycosis, ncnc anemia, thyroid dysfunction, recurrent UTI, s/p sepsis (Enterobacter) faecium)	acute/ early	DAIR	yes	79	polymicrobial	4	Infection control impossible in spite of repeated radical surgical interventions and antibiotic/antimycotic treatment.
**3**	84	m	26.8	no	no	3	Myelodysplastic syndrome with anemia; immunosuppressive therapy with Azacitin and steroids; unclear lung disease (Asbestosis?)	acute/ early	staged revision arthroplasty	yes	88	strept. sp.	2	Infection control impossible in spite of TKA-explantation and antibiotic treatment. Sepsis. Involvement of lower leg with non-vital musculature.
**4**	54	m	25.8	yes	yes	2	indication for TKA: open tibial plateau fracture with neurological compromise (Gustilo 3C), treated with megaprosthesis myocutaneus flap andinterpolated gastroc/soleusflap	acute/ early	AKA	yes	128	anaerobic bact.	3	Megaprothesis. Arthrodesis with fibula autograft considered but patient prefers AKA in favour of maintaining the integrity of the contralateral limb.
**5**	74	f	43.8	no	no	3	AH, sick-sinus-syndrome, CRI, inguinal mycosis, recurrent PE, COPD, CVI, ncnc anemia	acute/ early	staged revision arthroplasty	yes	101	CNS	8	Infection control impossible. Long term antibiotic suppression therapy badly tolerateds. Extensor mechanisme insufficient. Low demand patient.
**6**	88	f	18.6	yes	no	3	DM, iron deficiency anemia, carotis bifurcation stenosis, PAOD, CVD, CHF, AF, recurrent UTI	acute/ early	DAIR	yes	93	*S*. *aureus*	5	Infection control impossible in spite of repeated radical surgical interventions and antibiotic/antimycotic treatment. PAOD.
**7**	49	m	22.5	yes	no	3	HIV, Hepatitis A/B/C, polytoxicomania, tricuspid insufficiency, COPD, beta thalassemia, bilateral pneumonia	acute/ early	AKA	yes	89	*C. albicans*	8	Failed arthrodesis. Continued infection.
**8**	72	f	23.3	no	no	3	RA treated with Methotrexate and Humira; anticoagulation for recurrent PE; bilateral AKA for PJI	acute/ early	DAIR	yes	98	polymicrobial	21	Infection control impossible in spite of repeated radical surgical interventions and antibiotic treatment. Pain.
**9**	49	m	22.8	yes	yes	3	epilepsy, gastric ulcer, s/p postoperative MOF	acute/ early	DAIR	yes	98	polymicrobial	17	Infection control impossible in spite of repeated radical surgical interventions and antibiotic treatment. Persistant intraarticular bleeding due to coagulation disorder.
**10**	62	m	21.1	yes	no	3	DM, AH, s/p middle lobectomy for pulmonary squamous cell carcinoma, CVI, steroids for polyarthralgia, lingual carcinoma, GERD	chronic/ delayed	staged revision arthroplasty	yes	77	polymicrobial	14	Persistent fistulating osteomylitis in spite of repeated radical surgical interventions and antibiotic treatment.
**11**	71	f	23.3	no	no	3	RA treated with Methotrexate and Humira; anticoagulation for recurrent PE; bilateral AKA for PJI	acute/ early	DAIR	yes	114	strept. sp.	24	Infection control impossible in spite of repeated radical surgical interventions and antibiotic treatment. Pain.

Patients underwent between 2 and 24 surgeries in total. Three cases went directly on to AKA. In five cases the initial treatment strategy consisted of DAIR, in three cases of staged revision arthroplasty. The respective indication for amputation was uncontrollable local and/or systemic infection, often in combination with mechanical problems such as insufficient bone stock or chronic dislocation, or with concomitant direct influencing factors such as peripheral arterial occlusive disease (PAOD). In each individual case, AKA was considered as a last resort.

## Discussion

With the ageing of society as well as the advances of modern medicine and modern orthopedics, the volume of joint replacements that are performed has risen tremendously and will continue to increase [28, 29]. Inevitably, the number of complications to treat will augment simultaneously. PJI is one of the most common complications necessitating re-intervention and is associated with high morbidity and mortality. A recently published article by Kurtz et al. found PJI to be associated with a 5-year overall survival of only 71.7% for the knee, and 67.2% for the hip, respectively, putting PJI in the second leading place overall in terms of mortality rate, surpassed only by cancer [1].

Treatment of PJI aims to eradicate the infection while salvaging life and limb. In very few cases, though, therapy may fail in spite of adequate intervention, which in most cases comprises (staged) revision arthroplasty alongside pathogen-directed antibiotic coverage. In this event, the otherwise in the context of trauma applied principle of “life before limb” may have to be extended to the situation of PJI and infection control by addressing the source of infection via AKA may have to be considered. George et al. [30] recently published that the incidence of AKA related to PJI in the USA almost quadrupled between 1998 and 2013. Notably, AKA in this context multiplies mortality [15, 31]. Furthermore, low functional status has to be anticipated with at best half of the surviving patients reaching the ability to walk [14, 15].

As a university hospital providing subspecialized orthopedic tertiary care we often treat complex patients both in the context of primary arthroplasty but also in revision and referral situations. This may explain the fact that our amputation rate in PJI cases has been higher than previously reported [13]. In each individual case, the indication for AKA in this context was made as a last resort and after interdisciplinary consultation and discussion with the patient and/or their family. In view of the subject matter’s grave implications and its relevance to a growing number of patients, this study was conducted with the objective to find out whether or not there are patient- or treatment-associated elements that would indicate those patients at risk of ending up with AKA. And if so: are these elements accessible to treatment or could their recognition otherwise change our medical decision-making?

Previously published literature concentrated on identifying variables that are commonly associated with the development of PJI. Several comorbidities have been found linked to PJI, i.e. chronic cardiovascular, pulmonary and renal conditions, preoperative anemia, diabetes, depression and psychoses, obesity, rheumatologic disease, metastatic tumor, male gender, and higher comorbidity scores [1, 19, 32], but also the exposure to previous surgery [33]. Furthermore, measures such as antibiotic prophylaxis, surgical site preparation and operating room environment, improved control of post-operative glycaemia, appropriate management of malnutrition, preoperative anemia, and smoking cessation have been proven to minimize risk of PJI [1, 3, 19, 34]. Nevertheless, PJI cannot always be avoided and we then find ourselves having to treat complex patients presenting this challenging complication. Using the earlier mentioned protocols, treatment is in most cases successful. Publications concerning risk factors for PJI treatment failure, and specifically influencing factors of AKA in the context of PJI, are sparse. Kurtz et al. identified male gender, heart disease and higher Charlson comorbidity index as the most strongly associated patient related factors with PJI treatment failure [1]. Son and colleagues’ results overlapped partially with these findings. Yet, his group proposed that economical motives, region of residence, as well as surgical volume of the respective institution further played a role [13].

This study demonstrates that there were few patients with minimal comorbidities who ended up with amputation. Comparative analysis of the distribution of ASA scores yielded significant results in this respect. Due to the paucity of cases, however, the absolute reliability of our figures must be regarded with caution. For reasons of statistical feasibility we looked particularly at all cases classed ASA 2 and 3 – in other words patients with mild systemic disease without functional limitations and patients with severe systemic disease presenting functional limitations. While only one out of 27 ASA 2 patients failed limb salvage (4%), 10 out of 36 ASA 3 patients (28%) did. According to odds ratio ASA 3 patients were almost 12 times more likely to experience AKA than the rest. Comparison of the distribution of ASA scores between the AKA and the LS subgroup using Pearson Chi-square test was also significant (*p* = 0.009). Notably, no statistic difference was found when using the Charlson comorbidity index, another tool that is commonly used for risk stratification in orthopedic surgery [13, 25, 35, 36]. While the discrepancy of our results concerning ASA and Charlson score was somewhat surprising, we learned that other authors have made similar observations. Various researchers have stated that the ASA system may be a better predictor for adverse events than the Charlson score [37, 38] which, despite its formulaic collection of number and severity of conditions, seems to lack clinical applicability.

Alcohol abuse was identified as an additional statistically significant variable. While this seems logical, the validity of this finding may be questioned due to the low number of patients. For the same reason – lack of statistical validity - an analysis of the individual conditions summarized in the above-mentioned scores was not carried out.

Within our patient collective, 100% of AKA cases were anemic at time of admission to hospital for treatment of PJI, whereas only 58% of the LS patients (*p* = 0.022) presented hemoglobin parameters inferior to the threshold values defined by the World Health Organization (WHO) which are 120 g/dl for women and 130 g/dl for men [26]. The average hemoglobin was 99.9 ± 15.1 g/dl in the amputees, as opposed to 118.2 ± 19.9 g/dl in the controls (*p* = 0.011). Etiologically, we assume anemia of chronic disease to be the most common type of anemia amongst the studied patient collective. This assumption applies with regard to both groups. Differentiated laboratory testing for sub-diagnosis of anemia was not performed. A correlation to the duration of symptoms of infection was not demonstrated and the duration of symptoms did not significantly differ between the two groups. Notably, preoperative anemia has previously been found to increase the risk to develop PJI in patients undergoing total joint replacement [39]. Subsequently, a 2017 study by Lu et al. demonstrated - by means of a multivariate regression model directed to evaluate the effect of anemia in the context of septic revision surgery - that anemia was associated with an increased risk of complications, amongst them persistence of local deep infection, sepsis and septic shock [40]. As described above, treatment of these septic complications may, in turn, sometimes necessitate AKA in order to eliminate the primary infection site. Thus, our study illustrates one of the possible consequences of Lu’s findings and thereby underpins his conclusion to regard preoperative anemia as an important clinical risk factor in patients with PJI. We deduce that early recognition of the problem will allow for timely introduction of adequate causative treatments such as supplementation of vitamins, iron, or erythropoietin, amelioration of comorbidities that are commonly associated with anemia, and/or allogeneic blood transfusions [41]. Nevertheless, at this point we can neither imply that better pre- and perioperative blood management would have a positive impact on the outcome, nor that non-responding to treatment of anemia in patients with PJI could be an indicator of limb salvage failure.

On a qualitative level, it should be remarked that some of the amputees presented circumstances of sorts that may have rendered them particularly prone to infectious complications. Immunosuppression, as a consequence of disease and/or immunosuppressive therapy was, whilst no specific scoring was applied, widely observed amongst the AKA patients. Named by way of example, there was a polytoxicomaniac with HIV and hepatitis A/B/C in whom a monomicrobial PJI caused by *C. albicans* was diagnosed. As has been found, drug abuse is a predisposing factor for fungal PJI [42]. Moreover, as we have previously published, patients with illicit drug abuse and concomitant HIV and/or hepatitis present a catastrophic incidence of AKA and arthrodesis in case of knee PJI [43]. Several other patients were severely immunocompromized, one of which acutely with myelodysplastic syndrome which was diagnosed shortly after TKA implantation, and was treated by Azacitidine and steroids (*3*). By the time of presentation at our institution, this patient was septic and his infection had extensively disseminated from the prosthetic knee joint into the soft tissues of the lower leg leading to non-vital musculature and thus the impossibility of local infection control. Speaking of which, the importance of the integrity of the periarticular soft tissues in the context of knee joint replacement and treatment of PJI has been reported before; regarding this subject, authors have described poor wound conditions, preexisting scars or dystrophic skin as relevant risk factors for potentially devastating complications including the need for amputation [44, 45]. In our patient cohort, *case 4*, a young patient, who had initially received primary plastic reconstruction simultaneously with the implantation of a megaprothesis for treatment of an open fracture, and who continued to have bad soft tissue conditions, exemplifies this.

With respect to additional surgeries, we found that the frequency differed between AKA and LS patients. Statistical analysis showed a trend towards an elevated number of minor reoperations involving the skin and subcutaneous layer in the AKA group. Furthermore, we observed that the majority of the amputees had to endure an unreasonable seeming total number of surgeries - a median of eight, but up to 24 in one case. Five of them started out with an attempt of implant retention (DAIR) that later failed. In retrospect, the burden of first having to bear such a high number of surgeries in conjunction with intravenous antibiotic therapy in a hospital environment, and to then nonetheless having to undergo AKA, an intervention associated with a 1-year mortality of at least 50 to 60% [15–17, 31], seems very high. Our observations also prompt the consideration of whether DAIR is an adequate strategy for patients with severe comorbidities and immunocompromise, even in case of acute PJI. Such patients may be candidates for being assigned to the staged revision arthroplasty route of treatment, regardless of the reported symptom duration. Motivated by both this formal investigation and our clinical experience, we henceforth consider the idea of AKA as a sort of definite treatment option at an earlier stage. Along the same lines, Khanna et al., who have assessed patient satisfaction following AKA for chronically infected TKA, recommended discussing the option of AKA after a maximum of six surgeries. His group had found a high percentage of satisfaction among their seven amputees; six of which would have chosen to have the amputation sooner, given the choice [18].

This study has a number of limitations. Firstly, the study population is relatively small and heterogeneous, while the amount of examined influencing factors is vast. This is, on the one hand, due to the fact that the prevalence of AKA for treatment of PJI remains a rarity despite its aforementioned increase. On the other hand, the inclusion of a large spectrum of parameters was indispensible by reason of the complexity of the affected patients. While statistic significance and trend should therefore be viewed with some reservation, our numeric results appear nonetheless corresponding to clinical observations, clinical experience and common sense. Beyond statistics, we were also able to offer detailed qualitative clinical information and laboratory results. Consequently, we consider our results valuable to future treatment of PJI patients. A multi-center study with a larger study population could allow for confirmation of our conclusions. Secondly, we were not able to enclose the entirety of patients treated for PJI at our institution during the examination period due to incomplete patient records in the earlier years. While we included AKA from 2005 to 2015, the LS control group consisted of patients from 2009 to 2015. Hence, no prevalence of successful revision TKA, versus joint fusion, versus AKA was recordable. Thirdly, as a result of the study’s retrospective nature, the accuracy of our data is limited to the information recovered from the institution’s medical records. Fourthly, no outcome scores for either patient group are available at present.

## Conclusion

Factors potentially influencing the outcome of knee PJI are diverse. Our analysis suggests that presence of severe comorbidities and immunosuppressive conditions, alcohol abuse, and preoperative anemia may increase the risk of PJI treatment failure resulting in the only available treatment option being amputation. Regarding clinical practice we draw the following conclusions:

First, we constitute that patients being investigated for suspicion of PJI, especially those without urgent indication for surgical intervention, should undergo differentiated testing for anemia early in the diagnostic process. Consequentially, preoperative causative treatments and/or allogeneic blood transfusions for correction of anemia can be considered.

Second, we acknowledge that, in this study population, almost all patients who ended up with AKA suffered of severe systemic disease and functional limitations, as represented by their ASA scores. Great value should therefore be attributed to the anesthesiologist’s preoperative evaluation of patients’ overall health. As these severely ill patients (ASA ≥ 3), especially those with immunosuppressive conditions or long-term medication, have high rates of treatment failure after DAIR, consideration to proceed to staged revision procedures should be made.

## Data Availability

All data relevant to this this study are included in this published article. The original datasets as extracted and processed are available from the corresponding author on reasonable request.

## Abbreviations

AKA: Above-knee-amputation
ASA: American Society of Anesthesiologists
CRP: C-reactive protein
DAIR: Debridement, antibiotics and implant retention
ESR: Erythrocyte sedimentation rate
HIV: Human immune deficiency virus
LS: Limb salvage
OR: Odds ratio
PAOD: Peripheral arterial occlusive disease
PJI: Periprosthetic joint infection
PMN: Polymorphonuclear neutrophil
TKA: Total knee arthroplasty
WHO: World Health Organization

## References

[1] Kurtz SM, Lau EC, Son M-S, Chang ET, Zimmerli W, Parvizi J (2018). Are we winning or losing the Battle with Periprosthetic joint infection: trends in Periprosthetic joint infection and mortality risk for the Medicare population. J Arthroplast.

[2] Kurtz SM, Ong KL, Lau E, Bozic KJ, Berry D, Parvizi J (2010). Prosthetic joint infection risk after TKA in the Medicare population. Clin Orthop Relat Res.

[3] Pulido L, Ghanem E, Joshi A, Purtill JJ, Parvizi J (2008). Periprosthetic joint infection: the incidence, timing, and predisposing factors. Clin Orthop Relat Res.

[4] Chen AF, Kinback NC, Heyl AE, McClain EJ, Klatt BA (2012). Better function for fusions versus above-the-knee amputations for recurrent Periprosthetic knee infection. Clin Orthop Relat Res.

[5] Robertsson O, Lidgren L, Sundberg M. The Swedish Knee Arthroplasty Register Annual Report 2017. Printed in Sweden 2017. Elvins Grafiska AB, Helsingborg. ISBN 978-91-88017-15-4.

[6] Choo KJ, Austin M, Parvizi J. Irrigation and debridement, modular exchange, and implant retention for acute Periprosthetic infection after Total knee Arthroplasty. JBJS Essent Surg Tech. 2019;9. 10.2106/JBJS.ST.19.00019. 10.2106/JBJS.ST.19.00019 PMC6974313 32051782

[7] Osmon DR, Berbari EF, Berendt AR, Lew D, Zimmerli W, Steckelberg JM (2013). Diagnosis and Management of Prosthetic Joint Infection: clinical practice guidelines by the Infectious Diseases Society of America. Clin Infect Dis.

[8] Parvizi J, Gehrke T, Chen AF (2013). Proceedings of the international consensus on Periprosthetic joint infection. Bone Joint J.

[9] Kuiper JWP, Vos SJC, Saouti R, Vergroesen DA, Graat HCA, Debets-Ossenkopp YJ (2013). Prosthetic joint-associated infections treated with DAIR (debridement, antibiotics, irrigation, and retention): analysis of risk factors and local antibiotic carriers in 91 patients. Acta Orthop.

[10] Kunutsor SK, Beswick AD, Whitehouse MR, Wylde V, Blom AW (2018). Debridement, antibiotics and implant retention for periprosthetic joint infections: a systematic review and meta-analysis of treatment outcomes. J Inf Secur.

[11] Nagra NS, Hamilton TW, Ganatra S, Murray DW, Pandit H (2016). One-stage versus two-stage exchange arthroplasty for infected total knee arthroplasty: a systematic review. Knee Surg Sports Traumatol Arthrosc.

[12] Pangaud C, Ollivier M, Argenson J-N (2019). Outcome of single-stage versus two-stage exchange for revision knee arthroplasty for chronic periprosthetic infection. EFORT Open Rev.

[13] Son M-S, Lau E, Parvizi J, Mont MA, Bozic KJ, Kurtz S (2017). What are the frequency, associated factors, and mortality of amputation and arthrodesis after a failed infected TKA?. Clin Orthop Relat Res.

[14] Fedorka CJ, Chen AF, McGarry WM, Parvizi J, Klatt BA (2011). Functional ability after above-the-knee amputation for infected Total knee Arthroplasty. Clin Orthop Relat Res.

[15] Sierra RJ, Trousdale RT, Pagnano MW (2003). Above-the-knee amputation after a Total knee replacement: prevalence, etiology, and functional outcome. J Bone and Joint Surg Am.

[16] Kristensen MT, Holm G, Kirketerp-Møller K, Krasheninnikoff M, Gebuhr P (2012). Very low survival rates after non-traumatic lower limb amputation in a consecutive series: what to do?. Interact Cardiovasc Thorac Surg.

[17] Rosen N, Gigi R, Haim A, Salai M, Chechik O (2014). Mortality and Reoperations following Lower Limb Amputations. Isr Med Assoc J.

[18] Khanna V, Tushinski DM, Soever LJ, Vincent AD, Backstein DJ (2015). Above knee amputation following Total knee Arthroplasty: when enough is enough. J Arthroplast.

[19] Bozic KJ, Lau E, Kurtz S, Ong K, Berry DJ (2012). Patient-related risk factors for postoperative mortality and periprosthetic joint infection in medicare patients undergoing TKA. Clin Orthop Relat Res.

[20] Lee J, Kang C-I, Lee JH, Joung M, Moon S, Wi YM, et al. Risk factors for treatment failure in patients with prosthetic joint infections. J Hosp Infect. 2010;75:273–76. 20635512

[21] Kheir MM, Tan TL, Higuera C, George J, Della Valle CJ, Shen M (2017). Periprosthetic joint infections caused by enterococci have poor outcomes. J Arthroplast.

[22] Jhan S-W, Lu Y-D, Lee MS, Lee C-H, Wang J-W, Kuo F-C (2017). The risk factors of failed reimplantation arthroplasty for periprosthetic hip infection. BMC Musculoskelet Disord.

[23] Parvizi J, Gehrke T (2014). Definition of periprosthetic joint infection. J Arthroplast.

[24] Doyle DJ, Garmon EH (2018). American Society of Anesthesiologists Classification (ASA class). In: StatPearls.

[25] Charlson ME, Pompei P, Ales KL, MacKenzie CR (1987). A new method of classifying prognostic comorbidity in longitudinal studies: developement and validation. J Chron Dis.

[26] World Health Organization. WHO: Haemoglobin concentrations for the diagnosis of anaemia and assessment of severity. Vitamin and mineral nutrition information system Geneva, world health Organization, 2011 (WHO/NMH/NHD/MNM/111). http://www.who.int/vmnis/indicators/haemoglobin.pdf. Accessed 12 Oct 2019.

[27] Kim PH, Leopold SS (2012). In brief: Gustilo-Anderson classification. [corrected]. Clin Orthop Relat Res.

[28] Cram P, Lu X, Kates SL, Singh JA, Li Y, Wolf BR (2012). Total knee arthroplasty volume, utilization, and outcomes among Medicare beneficiaries, 1991-2010. JAMA..

[29] Kurtz S (2007). Projections of primary and revision hip and knee Arthroplasty in the United States from 2005 to 2030. J Bone Joint Surg (American).

[30] George J, Navale SM, Nageeb EM, Curtis GL, Klika AK, Barsoum WK (2018). Etiology of above-knee amputations in the United States: is Periprosthetic joint infection an emerging cause?. Clin Orthop Relat Res.

[31] Schmiegelow MT, Sode N, Riis T, Lauritzen JB, Duus BR, Lindberg-Larsen M. Re-amputations and mortality after below-knee, through-knee and above-knee amputations. Dan Med J. 2018;65(12):A5520. 30511636

[32] Blanco JF, Díaz A, Melchor FR, da Casa C, Pescador D (2020). Risk factors for periprosthetic joint infection after total knee arthroplasty. Arch Orthop Trauma Surg.

[33] Tayton ER, Frampton C, Hooper GJ, Young SW (2016). The impact of patient and surgical factors on the rate of infection after primary total knee arthroplasty: an analysis of 64,566 joints from the New Zealand joint registry. Bone Joint J..

[34] Alamanda VK, Springer BD (2018). Perioperative and modifiable risk factors for Periprosthetic joint infections (PJI) and recommended guidelines. Curr Rev Musculoskelet Med.

[35] Elmallah RDK, Cherian JJ, Robinson K, Harwin SF, Mont MA (2015). The effect of comorbidities on outcomes following Total knee Arthroplasty. J Knee Surg.

[36] Lavelle EAD, Cheney R, Lavelle WF (2015). Mortality prediction in a vertebral compression fracture population: the ASA physical status score versus the Charlson comorbidity index. Int J Spine Surg.

[37] Ondeck NT, Bohl DD, Bovonratwet P, McLynn RP, Cui JJ, Shultz BN (2018). Discriminative ability of commonly used indices to predict adverse outcomes after poster lumbar fusion: a comparison of demographics, ASA, the modified Charlson comorbidity index, and the modified frailty index. Spine J.

[38] Gronbeck C, Cote MP, Lieberman JR, Halawi MJ (2019). Risk stratification in primary total joint arthroplasty: the current state of knowledge. Arthroplast Today.

[39] Greenky M, Gandhi K, Pulido L, Restrepo C, Parvizi J (2012). Preoperative anemia in total joint arthroplasty: is it associated with periprosthetic joint infection?. Clin Orthop Relat Res.

[40] Lu M, Sing DC, Kuo AC, Hansen EN (2017). Preoperative Anemia independently predicts 30-day complications after aseptic and septic revision Total joint Arthroplasty. J Arthroplast.

[41] Stauder R, Valent P, Theurl I (2018). Anemia at older age: etiologies, clinical implications, and management. Blood..

[42] Gebauer M, Frommelt L, Achan P, Board TN, Conway J, Griffin W (2014). Management of Fungal or atypical Periprosthetic joint infections. J Arthroplast.

[43] Bauer DE, Hingsammer A, Ernstbrunner L, Aichmair A, Rosskopf AB, Eckers F (2018). Total knee arthroplasty in patients with a history of illicit intravenous drug abuse. Int Orthop.

[44] Jones RE, Russell RD, Huo MH (2013). Wound healing in total joint replacement. Bone Joint J.

[45] Osei DA, Rebehn KA, Boyer MI (2016). Soft-tissue defects after total knee Arthroplasty: management and reconstruction. J Am Acad Orthop Surg.

